# Resting-State fMRI Functional Connectivity Alterations in Drug-Resistant Epilepsy Compared to Well-Controlled Epilepsy and Healthy Controls

**DOI:** 10.3390/neurolint18070129

**Published:** 2026-07-07

**Authors:** Petar Vasilev, Ekaterina Viteva, Anna Todeva-Radneva, Antonia Yaneva, Dora Zlatareva, Tina Zdravkova, Sevdalina Kandilarova

**Affiliations:** 1 Department of Neurology, Medical University of Plovdiv, 4002 Plovdiv, Bulgaria; eiviteva@abv.bg; 2 Department of Neurology, University Multi-profile Hospital for Active Treatment St. George—Plovdiv, 4001 Plovdiv, Bulgaria; 3 Department of Psychiatry and Medical Psychology, Medical University of Plovdiv, 4002 Plovdiv, Bulgaria; anni.todeva@gmail.com (A.T.-R.); sevdalina.kandilarova@mu-plovdiv.bg (S.K.); 4 Research Institute, Medical University of Plovdiv, 4002 Plovdiv, Bulgaria; zdravkovatina@gmail.com; 5 Department of Medical Informatics, Biostatistics and eLearning, Faculty of Public Health, Medical University of Plovdiv, 4002 Plovdiv, Bulgaria; yaneva.antonya@gmail.com; 6 Department of Diagnostic Imaging, Medical University Sofia, 1431 Sofia, Bulgaria; dorazlat@yahoo.com

**Keywords:** epilepsy, drug-resistant epilepsy, resting state, fMRI, functional connectivity

## Abstract

**Background/Objectives:** Epilepsy is a chronic brain disease characterized by recurrent epileptic seizures. It affects roughly 50 million people worldwide and around one third of the patients have drug-resistant epilepsy (DRE). The current study aimed to find differences in the whole-brain functional connectivity (FC) in patients with DRE compared to patients with well-controlled epilepsy (WCE) and healthy controls (HCs). **Methods:** This explorative, cross-sectional study included 92 participants (n_DRE_ = 30; n_WCE_ = 30; n_HC_ = 32) who underwent resting-state functional magnetic resonance imaging (fMRI). The CONN Toolbox was used to process and analyze the FC changes among the three groups. **Results:** There was a statistically significant increase of the FC between the left lateral prefrontal cortex, left inferior temporal gyrus (temporo-occipital), left lobules IV and V of the cerebellum and multiple cortical and subcortical structures in patients with DRE as opposed to WCE and HC. On the other hand, decreased FC was observed between three seeds (the posterior cingulate cortex, precuneus cortex, the right planum polare) and different frontal, temporal and occipital regions. Interestingly, the right nucleus accumbens (r_NAc) showed increased FC with the inferior frontal gyrus in DRE compared to WCE, whereas the r_NAc-left precentral gyrus FC was reduced in DRE as opposed to HC. **Conclusions:** The acquired information offers valuable insights into the neuronal networks associated with DRE. These data could be used for advancing diagnostic accuracy and future therapeutic strategies.

## 1. Introduction

Epilepsy is a chronic brain disease with diverse etiologies characterized by recurring epileptic seizures due to neuronal hyperexcitability and hypersynchronization. Affecting approximately 50 million people worldwide, epilepsy is one of the most common neurological conditions; around 30% of patients with epilepsy remain resistant to the applied proper medical treatment [1].

Poor seizure control leads to social distancing, secondary epileptogenesis, epileptic encephalopathy, increased risk for traumatism and sudden unexpected death in epilepsy. This directly influences the quality of life of both the patients and their families [2,3]. Exploring the anatomical changes and the pathophysiological mechanisms of drug-resistant epilepsy (DRE) is essential for better diagnosis and high-quality treatment.

In recent years, epilepsy has been increasingly understood not as a condition arising from a single focal point but as a disorder of complex and widespread neural networks. The development of non-invasive neuroimaging techniques, particularly functional magnetic resonance imaging (fMRI), has been pivotal in advancing this network-based perspective. By measuring brain activity through changes in blood oxygenation during the resting state, fMRI provides valuable insights into the altered functional connectivity (FC) in epilepsy. This technology is key in both exploring the pathophysiology of the disease and in the critical task of localizing the seizure onset zone for pre-surgical evaluation, thereby significantly impacting clinical management and patient outcomes [4,5,6].

The studies using fMRI during the resting state reveal significant changes in the connectivity between the major brain networks in patients with different types of epilepsy and healthy controls (HCs). FC is defined by synchronous signals registered simultaneously from spatially distant brain regions. This shows which brain regions are functionally connected, but it is unable to reveal the direction of interaction and how those regions influence each other (excitatory or inhibitory) [7,8]. Two of the most studied brain networks in epilepsy using fMRI are the default mode network (DMN) and dorsal attention network (DAN). The DMN is more active during passive rest, and it deactivates during a specific task [9]. The main brain structures of the DMN involve the posterior cingulate cortex, precuneus, medial prefrontal cortex and inferior parietal lobule [10]. It is believed that the DMN takes part in internal cognitive processes, like self-referring thoughts, autobiographic memory and future planning [11]. The dorsal attention network includes the intrapariteal sulcus and the frontal eye fields. It is responsible for voluntary goal-directed attention, such as voluntarily focusing on a specific task or location in space [12].

A study from 2021 described cortical and cortical-subcortical differences in FC between patients with mesial temporal lobe epilepsy, patients with benign epilepsy with centrotemporal spikes and HC [13]. Decreased FC within the DMN and DAN has been registered in epilepsy with generalized tonic-clonic seizures compared to HC [14]. Concerning DRE, significant alterations in the FC of DMN and DAN were documented between patients, and HCs [15,16]. Furthermore, differences in thalamo-hippocampal FC has been found between drug-resistant and well-controlled left temporal lobe epilepsy [17].

Despite these findings, there remains a lack of comprehensive knowledge on possible FC patterns differentiating DRE from well-controlled epilepsy (WCE) and HCs. The aim of this study was to apply an explorative approach using resting-state fMRI in order to define potential FC alterations in patients with DRE as compared to WCE and HCs.

## 2. Materials and Methods

### 2.1. Study Design and Participants

This cross-sectional study involved 110 participants between 18 and 55 years of age. A detailed history was taken from every patient about the time of seizure onset, epilepsy duration, seizure frequency, type of seizures, and number and type of medications used before and at the time of the study. Eligible patients were only those with epilepsy of unknown or genetic etiology who had been on a stable antiseizure medication regime for at least three months and maintained a strict seizure diary. Exclusion criteria involved patients with structural (tumor, trauma, cerebrovascular incident and/or malformation), infectious, metabolic, or immune-related epilepsy, individuals with contraindications for MRI, or those with decompensated somatic or psychiatric disorders that could interfere with the required examinations. All subjects scoring lower than 26 on the Mini Mental State Examination (MMSE) and higher than 14 on the Beck’s Depression Inventory (BDI) were also excluded from the research.

After a quality review of the neuroimaging data, 18 subjects were excluded due to excessive head movement (more than 0.9 mm); a final cohort of 92 subjects remained. The final sample of 92 people was divided into three groups: patients with DRE (*n* = 30), patients with WCE (*n* = 30), and HCs (*n* = 32). The subjects in both epilepsy groups were divided according to the criteria of the International League Against Epilepsy for DRE and WCE. The patients were recruited at the Neurology Department of UMHAT “St. George” Plovdiv, Bulgaria, and outpatient facilities, while the HCs were recruited from the community. Financial compensation was not provided to the participants for their involvement in the study.

The protocol of the research was approved by the Medical University of Plovdiv Ethical Committee and complied with Declaration of Helsinki. Each of the subjects provided written informed consent before participating in the study.

### 2.2. MRI Scanning Procedure

Each participant was scanned on a GE Discovery 750w 3T MRI system (General Elctric, Boston, MA, USA). The scanning protocol included (1) a high-resolution structural scan (Sag 3D T1 FSPGR) with TR (repetition time) = 7.2 ms, TE (echo time) = min/full, slice thickness = 1 mm, matrix size = 256 × 256, and flip angle = 12°; (2) a resting-state 2D Echo Planar Imaging (EPI) sequence with a slice thickness of 3 mm, TE = 30 ms, TR = 2000 ms, matrix size = 64 × 64, and flip angle = 90°. Before each functional scan, five dummy scans were acquired and consequently discarded. The instruction for the functional sequence was for the patients to remain as still as possible and to let their mind wander.

### 2.3. Statistical Analysis

All continuous variables were examined for normality. Group differences in demographic and clinical variables were assessed using one-way analysis of variance (ANOVA), with Tukey post hoc tests for continuous variables and Pearson’s chi-square tests for categorical variables.

For epilepsy-related variables (age at seizure onset and disease duration), group differences between WCE and DRE were assessed using independent-sample *t* tests.

Statistical significance was set at *p* < 0.05. All statistical analyses were performed using SPSS version 23.

### 2.4. Image Processing

The functional images were analyzed with the CONN toolbox (https://doi.org/10.1089/BRAIN.2012.0073) (http://www.nitrc.org/projects/conn (accessed on 18 December 2025) running under MATLAB R2022a on a Windows platform. The preprocessing pipeline comprised realignment with unwarping, correction for slice-acquisition timing, automated detection of outlier volumes, segmentation and normalization of both functional and structural images, and spatial smoothing using a Gaussian kernel with 8 mm full width at half maximum.

Denoising was then carried out by regressing out several nuisance covariates: five principal components from white matter, five principal components from cerebrospinal fluid, the six motion parameters and their first derivatives, motion outlier regressors (as implemented in CONN), and the effect of rest (two principal components) modeling the initial resting-state drift for each run.

For each participant, seed-based functional connectivity maps were generated for each seed from the toolbox atlas (the Harvard-Oxford atlas and the AAL atlas) and entered into second-level analyses in CONN. Group-level inferences were based on voxelwise parametric statistics derived from Random Field Theory, using a voxel-level threshold of *p* < 0.001 (uncorrected) combined with a cluster-extent threshold of *p* < 0.05 (cluster-level FWE corrected). Age and sex were included as covariates of no interest in the between-group analysis. The regions showing altered FC among all three groups are the following: the Posterior Cingulate Cortex (PCC), Lateral Prefrontal Cortex (LPFC), Inferior Temporal gyrus temporooccipitalis (ITG-TOC), Precuneus Cortex (PCU), Planum Polare (PP), Nucleus Accumbens (NAc), and Cerebellum 4-5 (CER).

## 3. Results

### 3.1. Demographic and Clinical Characteristics

A one-way ANOVA revealed significant group differences in age. Post hoc Tukey tests indicated that patients with DRE were significantly older than HCs, whereas no significant differences were observed between WCE group and HCs or between the two epilepsy groups. Sex distribution did not differ significantly across groups.

Independent-sample *t* tests indicated that patients with DRE had a significantly earlier age of onset than patients with WCE. Disease duration was also significantly longer in the DRE group compared with the WCE.

Statistically significant group differences were also observed for BDI and MMSE scores. Post hoc analyses showed significantly higher BDI scores in the DRE group compared with both the WCE group and HCs, while MMSE scores were significantly lower in both epilepsy groups relative to controls.

Seizure type data were available for all patients with epilepsy. Combined focal and generalized seizures were the most prevalent, followed by generalized seizures and focal seizures. Within-group analyses indicated that generalized seizures predominated in patients with WCE, whereas combined focal and generalized seizures were most frequent in patients with DRE (Table 1).

### 3.2. Functional Connectivity

**Figure 1:**
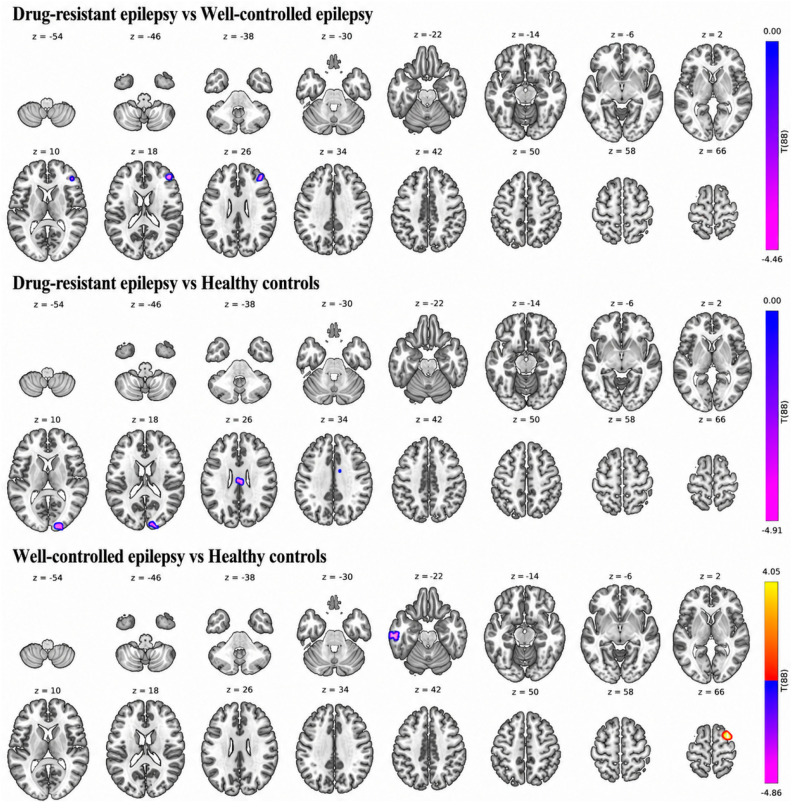
Between group differences in the resting-state functional connectivity of the posterior cingulate cortex (PCC). In patients with DRE, the PCC shows decreased connectivity with the right occipital lobe and the cingulate gyrus compared to HCs and reduced connectivity with the right frontal pole compared to the WCE group. In WCE as opposed to HC, the precuneus cortex demonstrates increased connectivity with the right superior and middle frontal gyri and decreased connectivity with the posterior areas of the left middle and inferior temporal gyri.

**Figure 2:**
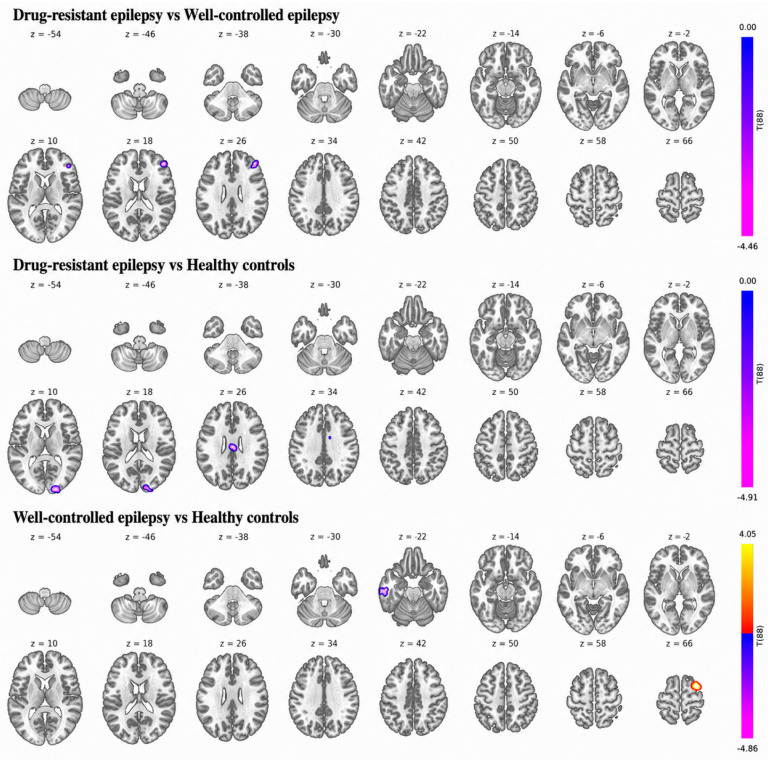
Between group differences in the resting-state functional connectivity of the precuneus cortex. In patients with DRE, the precuneus cortex shows decreased connectivity with the right occipital lobe and the cingulate gyrus compared to HCs and reduced connectivity with the right frontal pole compared to the WCE group. In WCE as opposed to HCs, the precuneus cortex demonstrates increased connectivity with the right superior and middle frontal gyri and decreased connectivity with the posterior areas of the left middle and inferior temporal gyri.

The between-group analysis revealed both increased (Table 2) and decreased (Table 3) FC between several seed regions and various cortical and subcortical areas. In patients with DRE, the posterior cingulate cortex (PCC) and the precuneus cortex presented decreased resting-state functional connectivity (rsFC) with the right occipital lobe and the cingulate gyrus compared to HCs and reduced rsFC with the right frontal pole compared to WCE. Accordingly, in WCE as opposed to HCs, PCC and the precuneus cortex showed increased rsFC with the right frontal gyri and decreased rsFC with the posterior areas of the left middle and inferior temporal gyri (Figure 1 and Figure 2).

**Figure 3:**
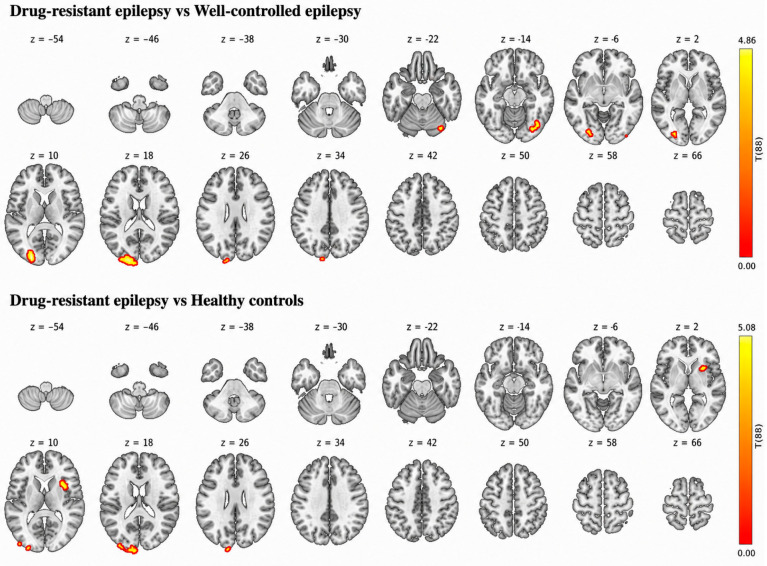
Between group differences in the resting-state functional connectivity of the left lateral prefrontal cortex (LPFC). In patients with DRE, the left LPFC shows functional hyperconnectivity with the left occipital pole and left lateral superior occipital cortex in comparison with the other two groups. In addition, in the DRE group the left LPFC demonstrates enhanced connectivity with the right putamen and right insular cortex as opposed to HCs and increased connectivity with the right occipital fusiform gyrus and right lateral inferior occipital cortex compared to WCE.

The left lateral prefrontal cortex (LPFC) in DRE exhibited functional hyperconnectivity with the left occipital pole and left lateral superior occipital cortex in comparison with the other two groups. Furthermore, the left LPFC demonstrated enhanced rsFC with the right putamen and right insular cortex as opposed to HCs, and increased rsFC with the right occipital fusiform gyrus and right lateral inferior occipital cortex as opposed to WCE (Figure 3).

**Figure 4:**
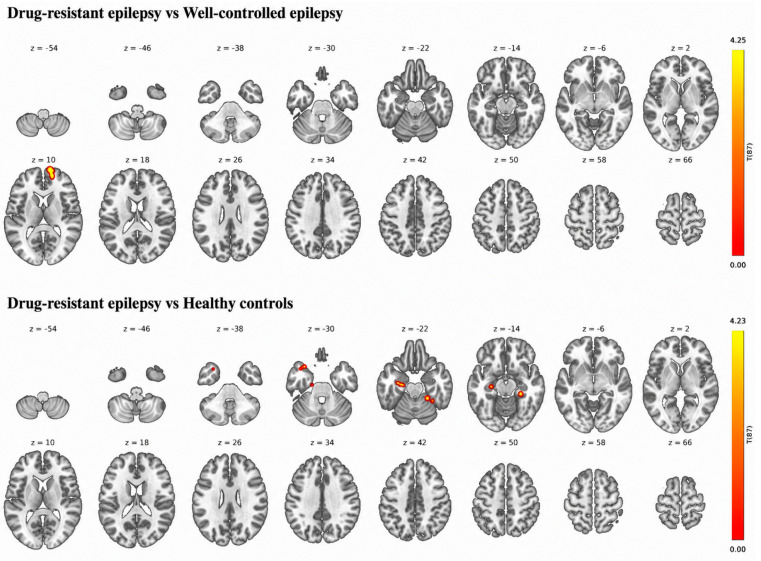
Between group comparison of the resting-state functional connectivity of the left inferior temporal gyrus (temporo-occipital). In patients with DRE, the left inferior temporal gyrus (temporo-occipital) demonstrates increased connectivity with the right and left parahippocampal gyri, right temporal occipital fusiform cortex, right IV and V cerebellum lobules, left hippocampus and left temporal pole in comparison with HCs. It also shows increased connectivity with the right frontal pole compared to WCE.

The left inferior temporal gyrus (temporo-occipital) in DRE displayed amplified rsFC with the right and left parahippocampal gyri, right temporal occipital fusiform cortex, right IV and V cerebellum lobules, left hippocampus and left temporal pole in comparison with HCs, whereas it also demonstrated increased rsFC with the right frontal pole compared to WCE (Figure 4).

**Figure 5:**
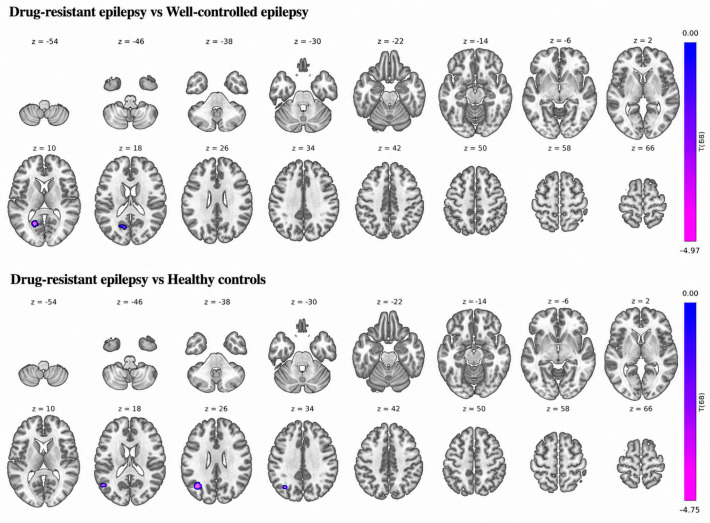
Between group comparison of the resting-state functional connectivity of the right planum polare. When compared to HCs, the DRE group displays hypoconnectivity between the right planum polare and both the left lateral occipital superior cortex and the left angular gyrus. In comparison to the WCE group, the right planum polare in DRE patients shows reduced connectivity with the precuneus.

In DRE, the right planum polare showed hypoconnectivity with the left lateral occipital superior cortex and left angular gyrus as opposed to HCs, and with the precuneus as opposed to WCE (Figure 5).

**Figure 6:**
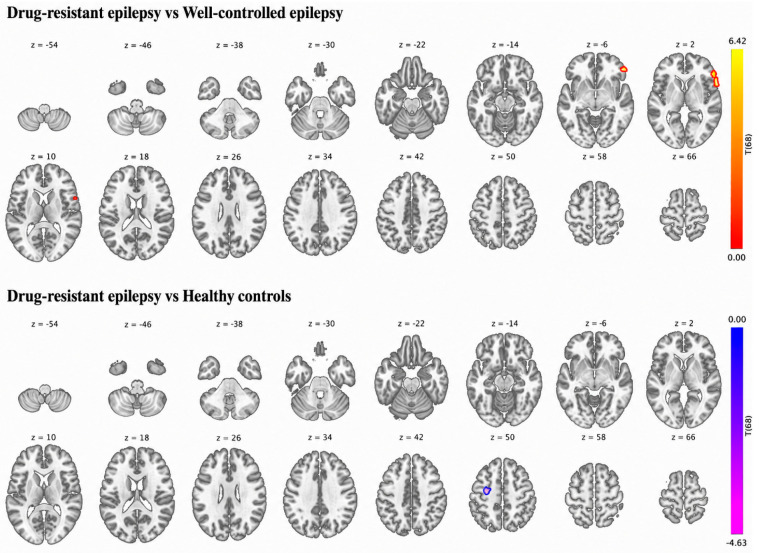
Between group comparison of the resting-state functional connectivity of the right nucleus accumbens. In individuals with DRE, the right nucleus accumbens demonstrates a significant reduction in connectivity with the left precentral gyrus when compared to HCs. When compared to the WCE group, the DRE group shows hyperconnectivity between the right nucleus accumbens and the right frontal pole (pars opercularis) and the right inferior frontal gyrus (pars triangularis).

The analysis revealed also that the right nucleus accumbens in DRE displayed reduced rsFC with the left precentral gyrus compared to HCs, while it exhibited hyperconnectivity with the right frontal pole and right inferior frontal gyrus (pars opercularis and pars triangularis) in comparison with WCE (Figure 6).

**Figure 7:**
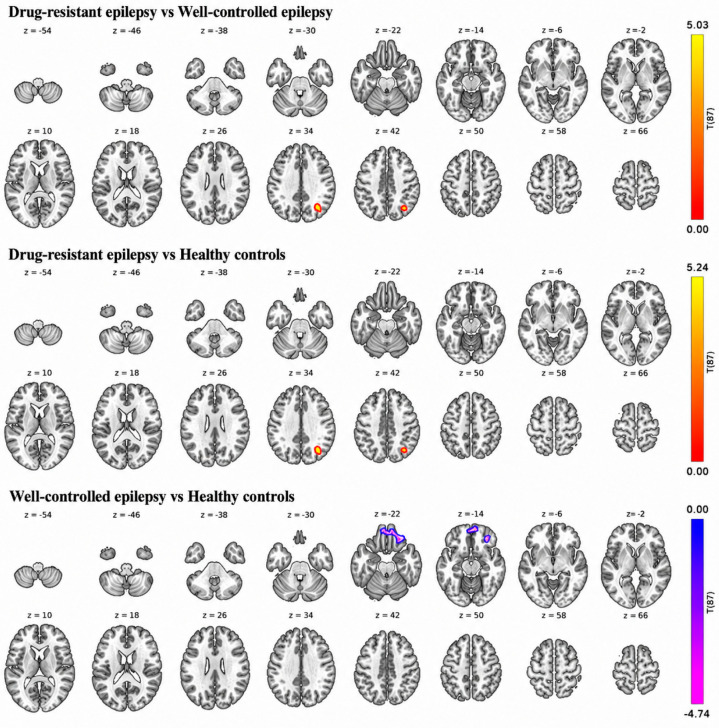
Between group comparison of the resting-state functional connectivity of the left IV and V lobules of the cerebellum. The DRE group exhibits increased connectivity with the right superior lateral occipital cortex compared to both HC and WCE patients. Comparing WCE to HCs, the same cerebellar regions show hypoconnectivity with right frontal pole, frontal medial cortex, right frontal orbital cortex and left frontal pole.

The left IV and V lobules of the cerebellum demonstrated enhanced rsFC with the right superior lateral occipital cortex for DRE as opposed to HCs and WCE. The results also revealed hypoconnectivity in the same region with the right frontal pole, frontal medial cortex, right frontal orbital cortex and left frontal pole in WCE compared to HCs (Figure 7).

## 4. Discussion

The current cross-sectional study explored the resting-state FC by means of seed-based analysis in patients with DRE, patients with WCE, and HCs. We found several brain regions in which FC significantly differs in DRE compared to the other two groups.

The first two regions with significantly different FC, namely the posterior cingulate cortex and precuneus cortex, are a part of the DMN. FC differences involving the PCC and/or precuneus cortex have also been reported between temporal lobe epilepsy and HCs [18], absence epilepsy and HCs [19], focal and generalized epilepsy [20], DRE and HCs [15], and left and right temporal lobe epilepsy [21].

The results underline the pivotal role of these two regions in the complex brain network alterations found in epilepsy. Both regions have multiple brain connections with different brain areas and are exclusively included in internal cognitive functions leading to decision making. Furthermore, there are reports on the favorable outcome of surgical treatment in posterior cingulate epilepsy [22,23].

Temporal lobes are brain areas with a low epileptogenic threshold that could potentially be a source of various types of seizures [24]. In our study, two temporal regions showed altered rsFC in DRE in comparison with WCE and HCs—the left inferior temporal gyrus (temporooccipital part) and right planum polare. The planum polare is located in the superior temporal lobe just in front of the Heschl’s gyrus. It is believed to be a part of the auditory cortex, responsible for speech and music processing, without further information concerning brain connections and its role in epilepsy. The temporo-occipital part of the inferior temporal gyrus is recognized for its involvement in advanced visual interpretation [25]. The results from the current research are in line with the fact that auditory and/or visual stimuli can provoke epileptiform activity and seizures. On the other hand, in 2020, Rafiee M et al. published a study that indicated listening to specific classical music may reduce seizure frequency, but the evidence was insufficient and the theory requires further investigation [26,27].

A common point about the PCC, precuneus cortex and inferior temporal gyrus is that all of these structures are not superficial, and epileptiform activity originating from them barely registers on a routine scalp EEG [22,28]. That is why their role may easily be underestimated in epileptogenesis and behavioral changes.

Another region showing distinct rsFC changes in patients with DRE is the left lateral prefrontal cortex. It is part of the executive control brain network (ECN), a network that is involved in executive functions, mostly when fast and adaptive control over movements during a specific task is required. Furthermore, ECN acts as a flexible mediator between the other resting-state brain networks and coordinates their interaction [11,29]. There have been studies showing that an epileptic seizure onset zone may have both local and remote effects in the networks beyond the lobe containing the focus. Such examples are the functional and metabolic changes of the frontal lobe in temporal lobe epilepsy (TLE) [30].

Neuropsychological tests have revealed frontal lobe cognitive dysfunction in patients with TLE, while fluorodeoxyglucose-positron emission tomography has shown decreased glucose metabolism in the frontal lobes of patients with drug-resistant TLE [31,32,33]. These data underly the interconnectivity changes between separate brain regions in epilepsy and their effect on cognition.

With regard to the FC alterations associated with epilepsy, an interesting structure that requires further attention is the nucleus accumbens. Studies in rodents demonstrated the role of the nucleus accumbens as a potential treatment target in TLE. Electrical lesion or pharmacological inhibition of the nucleus accumbens shell reduces seizure frequency in TLE in rats [34,35,36]. Furthermore, neurostimulation of the nucleus accumbens in several patients with DRE has also shown beneficial effect [37]. These findings correspond with our results, which indicate that the right nucleus accumbens has FC changes in patients with DRE compared to patients with WCE and HCs. The importance of nucleus accumbens is further supported by a study published in 2020, which revealed altered FC within this structure in patients diagnosed with left mesial temporal lobe epilepsy [38].

In recent years, significant scientific efforts have been focused on the cerebellum’s role in epilepsy and, more precisely, on its antiseizure effect. The cerebellum exerts inhibitory and excitatory effects on the cerebral cortex through complex neuronal circuits [39,40]. In epilepsy, these connections may serve as pathways for seizure propagation or as part of mechanisms suppressing seizure activity [28,41,42].

Moreover, consistent cerebellar abnormalities have been observed in epilepsy, including altered structural and FC, changes in blood perfusion, and volume reduction [43]. Studies showed that cerebellar atrophy has a positive correlation with the duration of the epilepsy and the frequency of the seizures [44]. Patients with temporal lobe epilepsy and patients with DRE are most susceptible to cerebellar degeneration [45]. Cerebellar atrophy is also reported in patients with sudden unexpected death in epilepsy and children with refractory epilepsy [46,47].

Furthermore, increased blood perfusion in the cerebellum has been registered in patients with epilepsy, and FC changes in the cerebellum have been reported in patients with TLE [48,49,50,51,52]. Our study further supports the involvement of the cerebellum in epilepsy, or more precisely, the altered FC in the left lobules IV and V in patients with DRE compared to patients with WCE and HC.

Because of its modulating effect on the cerebral cortex, the cerebellum stands at the center of high interest for treatment strategies in DRE. Experimental mice models showed that optogenetic or electrical stimulation of the cerebellum may have a positive effect on seizure control [53,54]. Electrical cerebellar stimulation for treating epilepsy in humans has been reported in many articles with ambiguous results, but with technical development and new promising findings, it is certainly a field worth exploring [55,56]. On the other hand, a study published in 2024 suggested that transcranial magnetic stimulation of the cerebellum has a positive outcome on seizure frequency in DRE [57].

There are several features that significantly distinguish the current research from previous resting-state fMRI studies on epilepsy. First, in the majority of prior studies, DRE was compared to either WCE or HCs [15,16,58,59]. Second, many studies examined the rsFC related only to predefined regions of interest (seeds) in the brain [58,60] or specific brain networks [18,19,59]. By contrast, in the present research, the data were acquired through a whole-brain rsFC evaluation and a comparison analysis between each of the three groups. This methodology allowed the identification of brain regions in DRE that demonstrate altered rsFC compared to both WCE and HCs.

While some studies have compared DRE, WCE, and HCs, their experimental designs differ considerably from the current approach. For instance, Pressl et al. (2018) focused specifically on TLE, identifying bilateral thalamo-hippocampal hypoconnectivity in drug-resistant TLE compared to well-controlled TLE, whereas well-controlled TLE and HCs showed similar FC patterns in the same regions [17]. In contrast, Pegg et al. (2021) employed graph theoretical analysis to evaluate the topological network organization between drug-resistant idiopathic generalized epilepsy (IGE), well-controlled IGE, and HCs. Although the IGE group as a whole demonstrated more regular interictal network topology and had a higher global connectivity compared to HCs, without alteration in hub node locations, no statistically significant differences were observed between the drug-resistant and well-controlled IGE subgroups [61].

## 5. Limitations

The present study has some limitations that need to be considered. One of the drawbacks is the heterogenous seizure types in the patient groups. Furthermore, given the relatively small sample size, we were unable to include more clinical characteristics as covariates due to a risk of overfitting. However, our findings may still be used as the basis for new hypotheses and are somewhat generalizable to a broader diagnostic group. Moreover, the cross-sectional design might be a disadvantage, as a longitudinal study would potentially yield more specific information on when and how FC changes appear in the course of the disease. A more comprehensive approach would involve following a group of HCs and patients with the same type of epilepsy from the onset of the diagnoses and over the course of several years. However, it should be considered that most of the fMRI studies in epilepsy share similar limitations.

## 6. Conclusions

The results from the study contribute to a better understanding of the pathological brain networks in DRE by identifying cortical and subcortical regions that demonstrate significant FC changes compared to WCE and HC. Future studies with larger and clinically homogeneous samples, longitudinal designs, and correlations to clinical outcomes are needed to determine whether the FC measures in the reported regions could serve as biomarkers of drug resistance or treatment targets in epilepsy.

## Figures and Tables

**Table 1:** Demographic and clinical characteristics of the study sample.

Variable	Healthy Controls (*n* = 32)	Well-Controlled Epilepsy (*n* = 30)	Drug-Resistant Epilepsy (*n* = 30)	Statistical Significance (*p*)
Age (years), *Mean* ± *SD*	28.84 ± 7.51	32.90 ± 9.32	35.80 ± 9.90	0.011 ^b^
Sex, n (%)				0.205 ^a^
Male	12 (37.5)	14 (46.7)	18 (60.0)	
Female	20 (62.5)	16 (53.3)	12 (40.0)	
Education, n (%)				
Primary or none	0	1 (3.3)	2 (6.6)	
Secondary	13 (40.6)	8 (26.7)	12 (40.0)	
Specialized secondary	0	6 (20.0)	10 (33.3)	
Higher education	19 (59.4)	15 (50.0)	6 (20.0)	
Age of onset (years), *Mean* ± *SD*	—	22.53 ± 10.17	17.00 ± 8.76	0.028 ^c^
Duration of epilepsy (years), *Mean* ± *SD*	—	10.43 ± 8.96	18.77 ± 10.75	0.002 ^c^
BDI, *Mean* ± *SD*	3.81 ± 4.43	5.60 ± 3.71	9.10 ± 4.58	<0.001 ^b^
MMSE, *Mean* ± *SD*	30.00 ± 0.00	28.67 ± 1.71	28.20 ± 1.42	<0.001 ^b^
Seizure type, n (%)				
Focal	—	6 (20.0)	7 (23.3)	
Generalized	—	18 (60.0)	2 (6.7)	
Combined focal and generalized	—	6 (20.0)	21 (70.0)	

Note: Values are presented as mean ± standard deviation. ^a^ Pearson chi-square test. ^b^ One-way ANOVA with Tukey post hoc comparisons. ^c^ Independent-sample *t* test between epilepsy groups only; BDI—Beck Depression Inventory; MMSE—Mini-Mental State Examination.

**Table 2:** Results from the between-group analysis demonstrating increased functional connectivity.

Seed	Between-Group Contrast	MNI Coordinates x,y,z	Cluster Size	Cluster Threshold	Regions Within the Cluster
DMN (PCC)	WCE > HC	+28+02+70	110	*p* < 0.05	Superior Frontal gyrus R
FrontoParietal LPFC L	DRE > WCE	−26−82+06	643	*p* < 0.001	Occipital Pole L, Lateral Occipital Cortex superior L
		+32+10+44	201	*p* < 0.05	Occipital Fusiform Gyrus R, Lateral Occipital Cortex inferior R
	DRE > HC	−10−100+22	266	*p* < 0.001	Occipital Pole L, Lateral Occipital Cortex inferior L
		+28+12+04	161	*p* < 0.05	Putamen R, Insular Cortex R
Inferior Temporal gyrus temporooccipitalis (to) L	DRE > WCE	+12+66+12	130	*p* < 0.05	Frontal Pole R
	DRE > HC	+26−34+16	120	*p* < 0.05	Parahippocampal Gyrus posterior R, Temporal Occipital Fusiform Cortex R, Cerebelum 4-5 R
		−20−16−26	116	*p* < 0.05	Hippocampus L, Parahippocampal Gyrus anterior L
		−38+12−34	113	*p* < 0.05	Temporal Pole L
Precuneus Cortex	WCE > HC	+30+06+66	147	*p* < 0.05	Superior Frontal Gyrus R; Middle Frontal Gyrus R
Accumbens R	DRE > WCE	+62+14+04	197	*p* < 0.001	Frontal Pole R, Inferior Frontal Gyrus pars opercularis R, Inferior Frontal Gyrus pars triangularis R
Cerebellum 4-5 L	DRE > WCE	+32−62+36	145	*p* < 0.05	Lateral Occipital Cortex superior R
	DRE > HC	+32−64+36	167	*p* < 0.05	Lateral Occipital Cortex superior R

DRE—drug-resistant epilepsy; WCE—well-controlled epilepsy; HC—healthy control; R—right; L—left; DMN—default mode network; PCC—posterior cingulate cortex; LPFC—lateral prefrontal cortex; Cerebellum 4-5—IV and V lobule of the cerebellum.

**Table 3:** Results from the between-group comparisons demonstrating decreased functional connectivity.

Seed	Between-Group Contrast	MNI Coordinates x,y,z	Cluster Size	Cluster Threshold	Regions Within the Cluster
DMN (PCC)	DRE < WCE	+46+44+22	123	*p* < 0.05	Frontal Pole R
	DRE < HC	+20−94+12	165	*p* < 0.05	Occipital Lobe R
		+2−18+28	140	*p* < 0.05	Cingulate Gyrus posterior, anterior
	WCE < HC	−58−22−22	139	*p* < 0.05	Middle Temporal Gyrus, post L
		+28+02+70	110	*p* < 0.05	Inferior Temporal Gyrus, post L
Precuneus Cortex	DRE < WCE	+46+44+22	181	*p* < 0.05	Frontal Pole R
	DRE < HC	+20−94+12	210	*p* < 0.05	Occipital Pole R
		+02−16+28	138	*p* < 0.05	Cingulate Gyrus posterior, anterior
	WCE < HC	−56−22−22	166	*p* < 0.05	Middle Temporal Gyrus, posterior L; Inferior Temporal gyrus, posterior L
Planum Polare R	DRE < WCE	−20−60+10	138	*p* < 0.05	Precuneous
	DRE < HC	−34−62+26	221	*p* < 0.001	Lateral Occipital Cortex superior L, Angular Gyrus L
Accumbens R	DRE < HC	−28−10+50	101	*p* < 0.05	Precentral Gyrus L
Cerebellum 4-5 L	WCE < HC	+32+28−14	688	*p* < 0.001	Frontal Pole R, Frontal Medial Cortex, Frontal Orbital cortex R, Frontal pole L

DRE—drug-resistant epilepsy; WCE—well-controlled epilepsy; HC—healthy control; R—right; L—left; DMN—default mode network; PCC—posterior cingulate cortex; Cerebellum 4-5—IV and V lobule of the cerebellum.

## Data Availability

The data supporting the findings of this study are available from the corresponding author upon request. However, due to privacy and ethical restrictions, raw patient data are not publicly available.

## Abbreviations

The following abbreviations are used in this manuscript:

BDI	Beck depression inventory
DAN	Dorsal attention network
DMN	Default mode network
DRE	Drug-resistant epilepsy
ECN	Executive control network
EEG	Electroencephalography
EPI	Echo planar imaging
FC	Functional connectivity
fMRI	Functional magnetic resonance imaging
HC	Healthy control
IGE	Idiopathic generalized epilepsy
L	Left
LPFC	Lateral prefrontal cortex
MMSE	Mini-mental state examination
MRI	Magnetic resonance imaging
PCC	Posterior cingulate cortex
r_NAc	Right nucleus accumbens
R	Right
rsFC	Resting-state functional connectivity
TE	Echo time
TLE	Temporal lobe epilepsy
TR	Repetition time
WCE	Well-controlled epilepsy
